# Practices participating in a dental PBRN have substantial and advantageous diversity even though as a group they have much in common with dentists at large

**DOI:** 10.1186/1472-6831-9-26

**Published:** 2009-10-15

**Authors:** Sonia K Makhija, Gregg H Gilbert, D Brad Rindal, Paul Benjamin, Joshua S Richman, Daniel J Pihlstrom, Vibeke Qvist, the DPBRN Collaborative Group

**Affiliations:** 1 Department of General Dental Sciences, School of Dentistry, University of Alabama, Birmingham, Birmingham, AL, USA; 2 HealthPartners Dental Group and HealthPartners Research Foundation, Minneapolis, MN, USA; 3 Private practitioner in Miami, FL, USA; 4 Division of Preventive Medicine, University of Alabama, Birmingham, Birmingham, AL, USA; 5 Permanente Dental Associates, Portland, OR, USA; 6 Department of Cariology and Endodontics, Royal Dental College, Copenhagen, Denmark

## Abstract

**Background:**

Practice-based research networks offer important opportunities to move recent advances into routine clinical practice. If their findings are not only generalizable to dental practices at large, but can also elucidate how practice characteristics are related to treatment outcome, their importance is even further elevated. Our objective was to determine whether we met a key objective for The Dental Practice-Based Research Network (DPBRN): to recruit a diverse range of practitioner-investigators interested in doing DPBRN studies.

**Methods:**

DPBRN participants completed an enrollment questionnaire about their practices and themselves. To date, more than 1100 practitioners from the five participating regions have completed the questionnaire. The regions consist of: Alabama/Mississippi, Florida/Georgia, Minnesota, Permanente Dental Associates, and Scandinavia (Denmark, Norway, and Sweden). We tested the hypothesis that there are statistically significant differences in key characteristics among DPBRN practices, based on responses from dentists who participated in DPBRN's first network-wide study (n = 546).

**Results:**

There were statistically significant, substantive regional differences among DPBRN-participating dentists, their practices, and their patient populations.

**Conclusion:**

Although as a group, participants have much in common with practices at large; their substantial diversity offers important advantages, such as being able to evaluate how practice differences may affect treatment outcomes, while simultaneously offering generalizability to dentists at large. This should help foster knowledge transfer in both the research-to-practice and practice-to-research directions.

## Background

Practice-based research networks (PBRNs) have been in existence in the United States since the 1970s [1]. The purpose of these networks is to join practitioners with academic researchers in developing and answering relevant research questions that can directly impact daily clinical practice [2]. PBRNs offer unique advantages both to research and quality improvement, and foster information sharing between practitioners [3-6].

Traditionally, clinical research projects have been conducted in academic settings. However, less than 1% of Americans receive their health care in that type of setting [7]. With studies initiated and developed by non-academic practitioners and conducted in their offices, results should be more relevant to these clinicians. Therefore, these studies should lead to improved clinical treatment in a shorter amount of time in these non-academic settings, as compared to conventional clinical research done in academic settings by academic dentists [6,8,9].

In the past, PBRNs have focused on non-dental areas, such as family medicine, internal medicine, pediatrics, and ophthalmology. Recognizing the success of physician-based PBRNs, the U.S. National Institute of Dental and Craniofacial Research (NIDCR) funded three oral health PBRNs in 2005, one of which is "DPBRN" (Dental Practice-Based Research Network). The purpose of these networks is to answer questions raised by dental practitioners in everyday clinical practice and to evaluate the effectiveness of current strategies to prevent, manage, and treat oral diseases and conditions [8,9]. DPBRN is unique in that it encompasses four regions in the United States and one in Scandinavia. The United States regions are: (1) the Alabama/Mississippi region (AL/MS), which almost entirely comprises dentists in private practice, although a few practices are in public health settings; (2) the Florida/Georgia region (FL/GA), which also comprises almost entirely dentists in private practice, although a few practices are in public health settings; (3) the Minnesota region (MN), which mainly comprises dentists employed by HealthPartners (HP), but which also has dentists in private practice in Minnesota; (4) the Permanente Dental Associates region (PDA), which is comprised entirely of dentists in Oregon and Washington in the PDA organization, in cooperation with the Kaiser Permanente Northwest (KPNW) Research Foundation's Center for Health Research; and (5) the Scandinavian (SK) region comprises dentists in Denmark, Norway, and Sweden, about one-half of whom are in private practice and one-half of whom are in a public health setting [6].

The regions comprising DPBRN were selected for various reasons and have been described previously [6]. The University of Alabama at Birmingham (UAB) is the administrative base for DPBRN and previously worked with Florida and Georgia [10]. The University of Florida conducted restorative dentistry practice-based studies in the past in Florida as well as Scandinavia [11,12]. Including Scandinavia in DPBRN adds to practitioner diversity within the network and helps to identify both preventive and restorative international variations in treatment, which can help identify research priorities for future DPBRN projects [6,13]. For two DPBRN regions, collaborations were established with two organizations: HP of Minneapolis, Minnesota, and PDA, of the greater metropolitan Portland, Oregon area. HP is a prepaid, multi-specialty group that provides comprehensive health care. The HP Dental Group is staffed by 58 dentists at 14 clinic locations that serve about 100,000 enrollees. PDA is a multi-specialty dental group that contracts with KPNW to provide dental services for KPNW prepaid comprehensive health plan members. PDA includes 117 dentists in 14 dental clinics in Oregon and Washington that serve about 180,000 members with dental benefits. The HP and PDA groups have conducted practice-based research, including joint collaborative projects [14,15].

In order to participate in DPBRN, practitioners must complete a DPBRN Enrollment Questionnaire. Dental practices are recruited in several ways. Licensed practitioners from the regions (AL/MS, FL/GA, MN, PDA, and SK) receive a mailing that describes DPBRN and invites them to participate. They are also recruited through DPBRN booths at dental meetings, at DPBRN orientation sessions, lectures given to dental students, as well as the public DPBRN website [6].

Recent findings from the DPBRN Enrollment Questionnaire are consistent with the conclusion that the DPBRN practitioner-investigators have much in common with dentists at large [16], meaning that results from DPBRN studies should be generalizable to the larger population of dentists. Although one objective of DPBRN is to comprise practices from which results should be generalizable to practices at large, another equally-important objective is that these practices also constitute a diverse range of practitioner-investigators, practice settings, patient populations, and geographic locations. This diverse range of practice settings should contain practices in private practice, public health practice, and preferred provider, managed care, or health maintenance organization settings.

Meeting the objective for geographic diversity not only requires practices from different regions nationally and globally, but also requires a mix of rural and urban locations. Therefore, although our earlier work documented that DPBRN practitioner-investigators have much in common with dentists at large [16], the objective of this current paper is to determine whether or not we met another DPBRN objective: to comprise a diverse range of practice settings and practitioners. Having substantial diversity offers important advantages, such as being able to evaluate how practice differences may affect treatment outcomes. We determined whether or not we met this objective by testing the hypothesis that there are statistically significant and substantive differences in key characteristics across DPBRN regions, based on responses to the DPBRN Enrollment Questionnaire from dentists who participated in DPBRN's first network-wide study.

## Methods

### The DPBRN Enrollment Questionnaire

Both dentists and dental hygienists can be DPBRN practitioner-investigator members. To become a member of DPBRN, practitioners must complete a 101-item enrollment questionnaire. This questionnaire queries information on practitioner characteristics, practice characteristics, and patient characteristics, and was largely taken from the Florida Dental Care Study [17]. The Enrollment Questionnaire is publicly-available at  under the "Enrollment/Join" tab. As of December 10, 2007, 1123 dentists had completed the questionnaire. Table 1 provides the distribution of participants who completed the enrollment questionnaire, by region. A total of 25 dental hygienists also had completed the questionnaire, but we excluded them from the current analyses because this paper focuses on dentist practitioner-investigators only. Table 2 provides a list of key practitioner, practice, and patient characteristics from the Enrollment Questionnaire, with an explanation of certain characteristics.

**Table 1:** Number of Dentists Participating in the Enrollment and Caries Questionnaires

**Number of Dentists Who Completed a DPBRN Enrollment Questionnaire, by Region**						
**Region**	**AL/MS**	**FL/GA**	**MN**	**PDA**	**SK**	**Total**

**Number**	822	123	54	64	60	1123
**Percent**	73	11	5	6	5	100.0

**Number of Dentists Who Completed a DPBRN Enrollment Questionnaire and Who Also Completed the DPBRN Caries Questionnaire, by Region (restricted to practitioners who perform at least some restorative dentistry procedures)**						

**Region**	**AL/MS**	**FL/GA**	**MN**	**PDA**	**SK**	**Total**

**Number**	306	106	32	51	51	546
**Percent**	56	20	6	9	9	100.0

AL/MS: Alabama/Mississippi

FL/GA: Florida/Georgia

MN: Minnesota

PDA: Permanente Dental Associates

SK: Scandinavian countries: Denmark, Norway, and Sweden

**Table 2:** Practitioner and Practice Characteristics Available From the DPBRN Enrollment Questionnaire

**Practice setting**	**Patient population**	**Dental procedure characteristics**	**Dentist individual characteristics**
Number of different sites at which you provide patient care at least once each week	Age distribution	Percentage of your time each day spent doing specific types of procedures ^3^	Gender
Whether you practice full-time or part-time	Racial distribution	Percentage of procedures that are done mainly for esthetic reasons	Race
Number of full-time dental hygienists in your practice	Percentage of revenues or charges that are derived from different payment sources ^2^	Percentage of certain procedures that you refer to other dentists ^4^	Year of graduation from dental school
Number of full-time dental assistants in your practice		Percentage of patients who get certain services at some time while they are patients in your practice ^5^	
Number of dental chairs you use regularly in your part of the practice		Number of root canal procedures that you do or refer each month	
Number of patient visits you personally have during a typical week		Number of dental extractions that you do or refer each month	
Typical number of days a patient has to wait for a new patient examination			
Typical number of days a patient has to wait for a treatment procedure appointment			
Practice busyness ^1^			

^1 ^Too busy to treat all people requesting appointments; Provided care to all who requested appointments, but the practice was overburdened; Provided care to all who requested appointments, and the practice was not overburdened; Not busy enough - the practice could have treated more patients.

^2 ^Dental insurance; Self-pay; Unpaid bills; Other.

^3 ^Percentage of patient contact time that you spend in a typical month performing the following procedures: Non-implant restorative dentistry; Implants (either implant surgery or time spent with implant placement); Dental extractions; Periodontal therapy (either time spent doing surgery or with non-surgical procedures); Endodontic therapy; Other (preventive and diagnostic).

^4 ^Percentage of the following procedures that you refer to other dentists: Periodontal surgery; Prosthetic crowns and bridges (other than implants); Implant surgery; Implant restorations; Anterior tooth root canals; Molar tooth root canals; Non-surgical extractions.

^5 ^Percentage of patients on which you or your staff perform the following procedures at some time while the patient is in your practice: Diet counseling; Blood pressure screening; Oral cancer screening examination; Oral hygiene instruction; Patient education from written pamphlets; Intraoral photographs; Intraoral video images taken; At-home whitening.

### The DPBRN Caries Questionnaire

The first network-wide study involving all five regions in DPBRN was entitled the "Assessment of Caries Diagnosis and Caries Treatment Questionnaire". This 10-page "Caries Questionnaire" inquired about which caries diagnostic and treatment procedures practitioner-investigators use, and how commonly they use them in their practices. Additionally, it posed certain clinical scenarios and respondents answered how they would recommend treating hypothetical patients in those scenarios.

A preliminary version of the questionnaire was administered to 16 DPBRN dentists to assess feasibility and comprehension of each questionnaire item. A subsequent pilot study finalized documentation of comprehension and item test-retest reliability across 15 days using a sample of 35 network dentists. All items in the final version met a test-retest reliability cutoff of kappa > 0.7.

To be eligible to complete the Caries Questionnaire, the practitioner-investigator must be a general dentist, pediatric dentist, or do at least some restorative dentistry, as well as have completed the DPBRN Enrollment Questionnaire. The questionnaires were mailed to the eligible practitioners, with second and third mailings sent to non-responders. One of the objectives of this study was to receive 200 responses to meet sample size requirements. Of the 970 DPBRN enrollees who were eligible, 546 completed the Caries Questionnaire, which well-exceeded expectations. Table 1 provides the distribution of participants who completed the Caries Questionnaire, by region.

The protocol was approved by the UAB, University of Florida, HP, PDA, and Scandinavian Institutional Review Boards and informed consent was explained and ascertained from each practitioner.

### Statistical Methods

To test the hypothesis that there are statistically significant differences across DPBRN regions with regard to dentist and practice characteristics, we used responses to the Enrollment Questionnaire made by practitioner-investigators who participated in the Caries Questionnaire. Analytic datasets were extracted, underwent a final quality-control analysis and were converted to SAS^® ^(SAS Institute, Cary, NC) and SPSS^® ^12.0 (SPSS, Inc, Chicago, IL) datasets. For categorical responses, chi-square goodness-of fit tests were used to test for differences between practitioners who completed the Enrollment Questionnaire and the Caries Questionnaire. Two-sample t-tests were used to examine continuous responses. For all cases, significance was determined by p < 0.05. Because some DPBRN responses were categorized into percentile ranges, a midpoint was assigned to each range in order to derive an approximate mean and standard deviation for reporting. We used this method for the variables regarding number of dental chairs, number of patients/week, number of days waiting for a new patient exam, and typical number of days a patient has to wait for a treatment procedure appointment. All analyses were done using SPSS^® ^12.0 and were independently verified using SAS^® ^9.1.

## Results

[Additional file 1-Table S1] presents the results comparing DPBRN practitioners who completed the Caries Questionnaire, by region.

### Practice setting

The number of different sites at which the practitioner provides care varied among the regions, with the MN and SK regions having the highest percentage of practitioners working at more than one location. These two regions also had the highest percentage of practitioners who do not practice full-time (19% and 30% respectively).

The number of full-time hygienists in the practice varied greatly across the regions. FL/GA (63%) and SK (72%) practices most commonly reported one full-time dental hygienist. MN and PDA practices had the highest percentage of three or more hygienists, due to the group practice structure in the PDA and HP practices.

The MN and SK practices had the highest percentages for having more than three full-time dental assistants, and the FL/GA practices had the highest mean number of dental chairs used regularly in their practices. Regarding the number of patient visits during a typical week, the AL/MS, FL/GA, and PDA practices most commonly reported 21-40 visits per week, while the MN and SK practices most commonly reported 41-60 visits per week. PDA practices reported the highest mean number of days that a patient has to wait for a new patient examination and a treatment procedure (29 days and 27 days; respectively). A high percentage of practitioner-investigators in all regions answered that they were able to provide care to all their patients, and were not overburdened.

### Patient population

The Enrollment Questionnaire divided patient ages into four groups: 1-18, 19-44, 45-64, and 65 years or older. Practitioner-investigators in all five regions most commonly reported that between 1% and 20% of their patients were between the ages of 1-18. For patients aged 19-44 and 45-64 years, practitioner-investigators from all regions most commonly reported that patients in this age group comprised between 21% and 40% of their patients. For all regions, most practitioners answered that patients aged 65 or older comprised 20% of their patients or less.

With regard to race of the patients, practitioner-investigators in the AL/MS, FL/GA, and PDA regions most commonly reported that 61-80% of their patients were white. MN and SK practitioner-investigators most commonly reported that between 81% and 100% of their patients were white. Practitioner-investigators in all regions most commonly reported that Black/African American patients comprised 20% of their patients or less.

With respect to payment sources, most of the SK practitioner-investigators reported that insurance comprises 20% or less of their practice's revenue; AL/MS and FL/GA practitioner-investigators most commonly reported that the figure was between 41% and 60% of their practice's revenue. MN and PDA practitioner-investigators most commonly reported that insurance comprised between 81% and 100% of their practice's revenue. In the self-pay category, MN and PDA practitioner-investigators most commonly reported that practice revenue coming from self-pay was 20% or less, which is because most of these practitioner-investigators practice in a HMO setting.

### Dental procedure characteristics

Although the Caries Questionnaire was limited to those who perform at least some restorative dentistry procedures, the amount of time devoted to performing non-implant restorative work nonetheless varied significantly across the regions. Practitioner-investigators in the MN region reported the highest percentage of time devoted to non-implant restorative procedures.

Most DPBRN practitioner-investigators spend 20% or less of their time placing or restoring implants, with a large percentage in the PDA region (77%) reporting that they do no implant procedures. There also were statistically significant differences between DPBRN regions and their practitioner-investigators with regard to the percentage of time they spend performing dental extractions, periodontal therapy, endodontic therapy, other procedures, and procedures done mainly for esthetic reasons [Additional file 1-Table S1].

With regard to referrals, practitioner-investigators in all DPBRN regions overwhelmingly responded that they refer between 81% and 100% of implant surgery, although practitioner-investigators in the SK region reported a significantly lower percentage. Practices in the AL/MS, FL/GA, and SK regions refer significantly lower percentages of their implant restorations, compared to practitioner-investigators in the MN and PDA regions. The PDA organization currently refers all implant restorations to dentists in their communities [Additional file 1-Table S1].

Although there were statistically significant differences between DPBRN regions, most practitioner-investigators refer 20% or less of their patients who need anterior root canal procedures. Referring molar root canal procedures is common among practitioner-investigators in the AL/MS, FL/GA, and PDA regions, but much less so among practitioner-investigators in the MN and SK regions. There were statistically significant differences between DPBRN regions with regard to the percentage of patients who are referred for non-surgical extractions [Additional file 1-Table S1].

Practitioners were asked questions regarding the percentage of their patients who receive certain services at some time while they are patients in their practice. There was substantial and statistically significant diversity across DPBRN practices. For example, when asked about diet counseling and blood pressure screening, the AL/MS, FL/GA, and SK practitioner-investigators responded that 20% or less of their patients receive this service, whereas these services were much more commonly provided in MN and PDA practices. Oral cancer screening and oral hygiene instruction services were very common in DPBRN practices, although even on these services, there was significant diversity among practices. Patient education from pamphlets was common, although again, there was significant diversity among DPBRN practices [Additional file 1-Table S1].

The use of intraoral photographs and video images was not common, although there was statistically significant and substantive diversity evident. Significant diversity was also evident with regard to the percentage of patients who are provided at-home tooth whitening.

The monthly practice volume of root canals and dental extractions - regardless of whether the DPBRN practitioner-investigator performed the procedure or whether it was referred to another dentist - also showed statistically significant and substantive diversity among DPBRN practices [Additional file 1-Table S1].

### Dentist individual characteristics

Most DPBRN practitioner-investigators are male, although there is significant diversity in this percentage across DPBRN regions. With regard to race of the practitioner-investigator, 100% of those in the Scandinavian region are white, in contrast to the other regions, which ranged from 80% to 94%. Practitioner-investigators of Asian descent comprise 14% of dentists in the PDA region.

There is substantial diversity within each DPBRN region as well as across regions with regard to the year in which the practitioner-investigator graduated from dental school. PDA has the youngest practitioner-investigators, with a plurality having graduated since 1994 [Additional file 1-Table S1].

Not shown in [Additional file 1-Table S1], DPBRN practitioner-investigators graduated from a broad range of dental schools. These include: University of Copenhagen, Denmark, Emory University, Georgetown University, Northwestern University, Oregon Health and Science University, School of Dentistry Malmö, Sweden, Tufts University, University of Alabama at Birmingham, University of Florida, University of Iowa, University of Michigan, University of Minnesota, University of Washington, and University of Oslo, Norway. This diverse group of dental schools, the full list of which includes many more dental schools, shows the wide range of didactic instruction that DPBRN practitioner-investigators have had.

## Discussion

In our previous DPBRN paper [16], we concluded that DPBRN practitioner-investigators as a group had much in common with dentists at large. The results in this current paper take the next analytic step and indicate that practitioner-investigators participating in DPBRN vary substantially with regard to many characteristics, including practice setting, patient population, dental procedure characteristics, and dentist individual characteristics. Previous research has shown that dental practice characteristics are related to the diagnostic services [18], preventive procedures [19], and treatment procedures [20,21] that patients receive, as well as to their treatment outcomes [17]. Establishing a PBRN with a diverse range of these practice characteristics creates a research context ripe for relating these characteristics to treatment outcomes and to success in moving new knowledge into routine practice.

Having a diverse group of individuals in DPBRN is important in that research topics come from members. Details regarding this process are provided elsewhere [6]. DPBRN receives input from members of the network at each step of the process. These ideas come from suggestions to our website , face-to-face meetings, orientation sessions, or visits to the practice during other studies. The ideas are then sent to the Executive Committee (EC), which has at least one practitioner-investigator representative from each region. Ideas for studies are discussed and prioritized by the EC, and if approved, are sent to the NIDCR to determine whether it overlaps with studies already funded by them. If not, DPBRN forms a protocol working group which includes at least one practitioner-investigator member. The idea is then formed into a full grant application, with the DPBRN practitioner-investigator member providing input to ensure that the study will be feasible and practical to conduct in a typical clinical practice.

Past studies have shown that it takes an average of 17 years to turn just 14% of original research findings into changes in care that will benefit patients [22]. When research is done in an academic setting, patients are not typically representative of the majority of those who receive care in a private setting, and the results of these studies may not be applicable to many communities [23-25]. PBRNs have great potential to speed this process [23,24,26-29]. If practitioners are involved with the development of the studies and are involved in projects that are relevant to everyday clinical practice (which is what DPBRN does), they are more likely to use the results [3]. An assessment of how applicable the findings of PBRN research are to the dental community at large is important in evaluating its impact on changing clinical practice [29]. With regard to DPBRN specifically, because ideas for almost all studies conducted by DPBRN originate from DPBRN practitioner-investigators themselves, DPBRN results have the potential to help shorten the delay before study results are incorporated into clinical practice [26]. In a 2000 ADA Membership Needs and Opinion Survey, 80% of ADA members felt that implementing treatment for their patients based on scientific findings was a 'critical' or 'significant' issue, stressing the importance of the findings from PBRNs [30].

These results do have some limitations. Of the 970 practitioners who received the Caries Questionnaire, 546 returned a completed questionnaire. Additionally, the majority of DPBRN practitioners who completed an Enrollment Questionnaire and the Caries Questionnaire were from the AL/MS region; 73.1% and 56.0% respectively. Although the necessary sample sizes for both questionnaires far exceeded a priori requirements, having a large number of respondents from one region also meant that this region necessarily contributed a higher percentage of respondents network-wide. DPBRN dentists, while coming from a diverse range of geographical areas and practice types, are not selected randomly. Similarly, those dentists who elected to participate in the first network-wide study are self-selected and therefore not random. Studies conducted in academic health centers also make no claims about the random selection of practitioners or patients in their studies, although characteristics of those practitioners and patients are always described. PBRNs improve upon the generalizability of their study results because "real-world" practitioners are the ones doing the studies, under "real-world" conditions, and with patients who enter the non-academic health care system. Our earlier paper demonstrated that, although PBRNs do not take random samples, DPBRN dentists had much in common with dentists at large, and in fact the only characteristic that was statistically different from the ADA survey data was the number of years since graduation from dental school [16]. This speaks to the generalizability of results from DPBRN studies. An additional next step is to demonstrate that participating practitioner-investigators comprise a diverse, broad range of backgrounds, practice environments, and patient populations, which also speak to the generalizability of results from DPBRN studies. The key value of the current study is that we are indeed able to demonstrate that diversity.

## Conclusion

Our objective was to determine whether we met our network's goal to engage a diverse range of practitioner-investigators in DPBRN research. We can conclude that our objective was indeed met because we were able to document statistically significant and substantive differences in key characteristics within and between DPBRN regions, based on responses to the DPBRN Enrollment Questionnaire from dentists who participated in DPBRN's first network-wide study. These results illustrated the diversity of DPBRN practitioner-investigators, their practices, and their patient populations. These findings demonstrate that a very broad range of dentists and practices are participating in DPBRN.

This diversity will allow us to relate key practice characteristics to treatment outcomes in DPBRN studies, and whether results from these studies can be successfully incorporated into routine dental practice in a broad range of practice settings. DPBRN practices simultaneously comprise a diverse range of characteristics while also having much in common with practices at large [22]. This within-network diversity and overall-network commonality to practices at large should increase the likelihood that DPBRN studies will be generalizable and applicable to other dental practices, thereby fostering knowledge transfer not only in the research-to-practice direction, but also in the practice-to-research direction.

## Competing interests

The authors declare that they have no competing interests.

## Authors' contributions

SKM drafted and edited the manuscript and also performed the statistical analysis. GHG drafted and edited the manuscript. DBR drafted and edited the manuscript. PB drafted and edited the manuscript. JSR drafted and edited the manuscript and also performed the statistical analysis. DJP drafted and edited the manuscript. VQ drafted and edited the manuscript. All authors read and approved the final manuscript.

## Pre-publication history

The pre-publication history for this paper can be accessed here:



## Supplementary Material

Additional file 1 **Practitioner and Practice Characteristics, in Percent, By Region, for the DPBRN Practitioners Who Completed the Caries Questionnaire**. The data provided represent the statistical analysis of the practitioner and practice characteristics of members who completed the first DPBRN study. Click here for file
