# Supplementary material for: Practices participating in a dental PBRN have substantial and advantageous diversity even though as a group they have much in common with dentists at large
Source: BMC Oral Health. 2009 Oct 15;9:26. doi: 10.1186/1472-6831-9-26 (PMC2768690; doi:10.1186/1472-6831-9-26)
Supplement: Additional file 1 — Practitioner and Practice Characteristics, in Percent, By Region, for the DPBRN Practitioners Who Completed the Caries Questionnaire. The data provided represent the statistical analysis of the practitioner and practice characteristics of members who completed the first DPBRN study. [file 1472-6831-9-26-S1.DOC]

**Table S1. Practitioner and Practice Characteristics, in Percent, By Region, for the DPBRN Practitioners Who Completed the Caries Questionnaire**

| **Characteristic** | **AL/MS**  **n=306** | **FL/GA**  **n=106** | **MN**  **n=32** | **PDA**  **n=51** | **SK**  **n=51** | **Total**  **n=546** | **P-Value** |  | | | | | | |
| --- | --- | --- | --- | --- | --- | --- | --- | --- | --- | --- | --- | --- | --- | --- |
| **Practice Setting** | | | | | | | | |  |  |  |  |  |  |
| Number of different sites at which you provide patient care at least once a week  1  2  3  >3 | 93  6  1  0 | 90  9  1  0 | 87  10  0  3 | 98  2  0  0 | 83  15  2  0 | 90  7  2  1 | **0.011** |  | | | | | | |
| Whether or not the practitioner practices full-time  Yes  No | 86  14 | 90  10 | 81  19 | 84  16 | 70  30 | 85  15 | **0.017** |  | | | | | | |
| Number of full-time dental hygienists in your practice  0  1  2  3  >3 | 3  36  40  12  9 | 15  63  15  2  5 | 4  7  29  21  39 | 0  2  2  96  0 | 7  72  21  0  0 | 5  37  30  19  9 | **<0.0001** |  | | | | | | |
| Number of full-time dental assistants in your practice  0  1  2  3  >3 | 3  43  37  10  7 | 4  24  52  9  11 | 0  6  23  13  58 | 0  4  96  0  0 | 6  26  35  9  24 | 3  32  44  9  12 | **<0.0001** |  | | | | | | |
| Number of dental chairs you use regularly in your part of the practice, mean (SD) | 5(2) | 10(5) | 6(5) | 4(3) | 3(2) | 5(3) | **<0.0001** |  | | | | | | |
| Number of patient visits you personally have during a typical week*  0-20  21-40  41-60  61-80  81-100  >100 | 7  37  33  14  6  3 | 6  47  29  10  3  5 | 3  38  53  6  0  0 | 0  53  45  2  0  0 | 5  19  51  21  4  0 | 6  39  36  12  4  3 | **0.002** |  | | | | | | |
| Typical number of days a patient has to wait for a new patient examination, mean (SD) | 9 (14) | 11(15) | 18(11) | 29(18) | 19(28) | 13(17) | **<0.0001** |  | | | | | | |
| Typical number of days a patient has to wait for a treatment procedure appointment, mean (SD) | 9(11) | 9(11) | 21(11) | 27(29) | 11(7) | 12(15) | **<0.0001** |  | | | | | | |
| Practice busyness  Too busy to treat everyone  Provided care to all, but overburdened  Provided care to all, not overburdened  Not busy enough | 10  18  56  16 | 3  16  56  25 | 20  27  50  3 | 8  16  66  10 | 22  33  41  4 | 10  19  55  16 | **<0.0001** |  | | | | | | |
| **Patient population** | | | | | | | | |  |  |  |  |  |  |
| Age distribution, in percent  1-18 year olds  0  1-20  21-40  41-60  61-80  81-100  19-44 year olds  0  1-20  21-40  41-60  61-80  81-100  45-64 year olds  0  1-20  21-40  41-60  61-80  81-100  65 or older  0  1-20  21-40  41-60  61-80  81-100 | 1  69  21  3  1  5  0  22  62  15  1  0  0  17  70  11  2  0  1  75  22  2  0  0 | 1  76  13  3  0  7  5  38  49  5  3  0  7  14  61  15  3  0  8  50  26  12  3  1 | 3  69  25  0  0  3  0  41  53  6  0  0  3  12  66  13  6  0  3  60  25  6  3  3 | 0  92  4  0  0  4  0  15  64  21  0  0  0  8  81  9  0  2  0  85  15  0  0  0 | 0  63  20  2  2  13  7  27  53  13  0  0  7  18  60  11  4  0  5  60  35  0  0  0 | 1  72  17  3  1  6  1  26  58  13  1  1  2  15  68  12  3  0  3  68  24  4  1  0 | **0.044**  **0.002**  **0.002**  **<0.0001** |  | | | | | | |
| Racial distribution, in percent  White  0  1-20  21-40  41-60  61-80  81-100  Black or African American  0  1-20  21-40  41-60  61-80  81-100 | 0  3  6  23  41  27  0  52  32  12  2  2 | 2  5  10  20  32  31  3  75  15  4  3  0 | 0  4  10  20  13  53  0  87  13  0  0  0 | 1  1  4  33  48  13  2  94  4  0  0  0 | 5  0  0  0  7  88  40  60  0  0  0  0 | 1  3  6  21  35  34  4  62  23  8  2  1 | **<0.0001**  **<0.0001** |  | | | | | | |
| Percentage of revenues or charges that are derived from different payment sources  Insurance  0  1-20  21-40  41-60  61-80  81-100  Self-Pay  0  1-20  21-40  41-60  61-80  81-100  Unpaid  0  1-20  21-40  41-60  61-80  81-100  Other  0  1-20  21-40  41-60  61-80  81-100 | 0  4  14  39  34  9    0  28  40  23  6  3  2  97  1  0  0  0  7  86  4  2  0  1 | 4  12  27  31  23  3    1  16  32  26  15  10  28  69  2  0  0  1  75  22  1  0  1  1 | 0  7  3  0  40  50    0  97  0  3  0  0  34  66  0  0  0  0  85  8  0  0  3  4 | 0  0  0  0  2  98  20  78  2  0  0  0  61  39  0  0  0  0  80  20  0  0  0  0 | 15  67  10  5  0  3    10  5  0  8  33  44  25  75  0  0  0  0  44  44  3  0  0  9 | 2  10  15  29  26  18    3  32  29  19  9  8  16  83  1  0  0  0  47  47  3  0  1  2 | **<0.0001**  **<0.0001**  **<0.0001**  **<0.0001** |  | | | | | | |
| **Dental procedure characteristics** | | | | | | | | |  |  |  |  |  |  |
| Percentage of your time each day spent doing specific procedures  Non-implant restorative (amalgams, composites, crowns, bridges, posts, foundations, etc.)  0  1-20  21-40  41-60  61-80  81-100  Implants (includes both prosthetic and surgical procedures)  0  1-20  21-40  41-60  61-80  81-100  Dental extractions (surgical and non-surgical)  0  1-20  21-40  41-60  61-80  81-100  Periodontal therapy (surgical and non-surgical)  0  1-20  21-40  41-60  61-80  81-100  Endodontic therapy (root canals and endodontic surgery)  0  1-20  21-40  41-60  61-80  81-100  Other (sealants, periodic and hygiene examinations, preventive dentistry, diagnostic, or other-specify)  0  1-20  21-40  41-60  61-80  81-100 | 0  5  16  34  34  11  1  97  2  0  0  0  0  94  6  0  0  0  0  100  0  0  0  0      0  91  8  1  0  0    1  85  12  1  1  0 | 0  4  19  37  33  7  15  83  2  0  0  0  6  80  11  2  1  0  18  81  0  0  0  1      12  76  11  1  0  0    3  77  14  4  1  1 | 0  0  3  38  50  9  7  93  0  0  0  0  3  97  0  0  0  0  10  90  0  0  0  0      0  97  3  0  0  0    0  85  15  0  0  0 | 0  0  12  55  31  2  77  23  0  0  0  0  0  90  8  2  0  0  32  66  0  2  0  0      4  94  2  0  0  0    0  94  6  0  0  0 | 0  12  32  29  17  10  12  81  5  2  0  0  7  93  0  0  0  0  7  72  19  2  0  0      5  88  7  0  0  0    10  64  15  8  3  0 | 0  5  17  37  32  9  13  85  2  0  0  0  2  90  6  2  0  0  9  88  2  1  0  0      3  89  8  0  0  0    2  82  13  2  1  0 | **0.047**  **<0.0001**  **<0.0001**  **<0.0001**  **<0.0001**  **0.012** |  | | | | | | |
| Percentage of procedures that are done mainly for esthetic reasons  0  1-20  21-40  41-60  61-80  81-100 | 1  71  18  7  2  1 | 2  62  20  12  2  2 | 0  93  7  0  0  0 | 6  94  0  0  0  0 | 5  84  11  0  0  0 | 1  73  18  6  1  1 | **<0.0001** |  | | | | | | |
| Percentage of certain procedures that you refer  Implant surgery  0  1-20  21-40  41-60  61-80  81-100  Implant restorations  0  1-20  21-40  41-60  61-80  81-100  Anterior root canals  0  1-20  21-40  41-60  61-80  81-100  Molar root canals  0  1-20  21-40  41-60  61-80  81-100  Non-surgical extractions  0  1-20  21-40  41-60  61-80  81-100 | 0  4  1  2  1  92  1  77  1  0  1  20  0  70  2  2  2  24  0  34  4  7  4  51  1  76  2  7  4  10 | 3  13  2  2  1  79  14  74  0  0  0  12  7  66  3  2  2  20  3  27  9  5  3  53  7  57  7  13  4  12 | 3  0  0  0  0  97  3  21  0  0  0  76  0  97  0  0  0  3  0  45  14  14  7  20  4  80  3  3  3  7 | 0  2  0  0  0  98  0  0  0  0  0  100  28  64  0  2  2  4  0  8  12  28  6  44  48  46  0  6  0  0 | 4  21  2  2  2  69  7  50  4  4  0  35  5  95  0  0  0  0  4  91  0  2  2  0  7  93  0  0  0  0 | 1  7  1  2  1  88  4  63  1  0  1  31  4  73  2  1  2  18  1  36  6  8  4  45  7  72  3  7  3  8 | **<0.0001**  **<0.0001**  **<0.0001**  **<0.0001**  **<0.0001** |  | | | | | | |
| Percentage of patients who get certain services at some time while they are patients in your practice  Diet counseling  0  1-20  21-40  41-60  61-80  81-100  Blood pressure screening  0  1-20  21-40  41-60  61-80  81-100  Oral cancer screening  0  1-20  21-40  41-60  61-80  81-100  Oral hygiene instruction  0  1-20  21-40  41-60  61-80  81-100  Patient education from pamphlets  0  1-20  21-40  41-60  61-80  81-100  Intraoral photographs  0  1-20  21-40  41-60  61-80  81-100  Intraoral video images  0  1-20  21-40  41-60  61-80  81-100  At-home tooth whitening  0  1-20  21-40  41-60  61-80  81-100 | 1  56  9  13  6  15    0  62  8  10  6  14  0  2  1  3  7  87  0  1  1  5  9  84  0  47  16  15  8  14    0  84  6  4  3  3  1  79  5  7  2  6  0  75  20  3  1  1 | 5  57  12  5  7  14  6  52  7  8  7  20  2  4  0  1  1  92  2  2  2  4  8  82  3  32  20  13  8  24    6  62  10  8  2  12  7  73  5  6  3  6  3  57  25  10  2  3 | 0  26  15  11  26  22  0  49  7  11  0  33  0  0  0  4  4  92  0  0  0  4  0  96  0  19  19  22  14  26  0  93  4  0  0  3  4  93  0  0  0  3  0  82  14  4  0  0 | 4  24  14  8  22  30  2  0  0  0  6  92  0  0  0  0  0  100  0  0  0  0  0  100  0  36  14  28  6  16  70  30  0  0  0  0  76  24  0  0  0  0  4  90  4  2  0  0 | 0  53  19  11  4  13  4  96  0  0  0  0  2  19  0  2  4  73  0  4  9  9  28  50  0  56  24  9  0  11  2  84  6  6  2  0  2  91  5  0  2  0  2  90  4  4  0  0 | 2  51  11  10  10  16  2  56  6  7  6  23  1  3  1  2  5  88  0  1  2  4  9  84  1  42  18  16  7  16  8  75  6  4  3  4  9  74  4  5  3  5  1  74  17  4  2  2 | **<0.0001**  **<0.0001**  **0.008**  **<0.0001**  **0.004**  **<0.0001**  **<0.0001**  **<0.0001** |  | | | | | | |
| Number of root canal procedures that you do or refer each month  0  1-5  6-10  11-15  16-20  >20 | 2  16  28  21  17  16 | 0  24  34  16  18  8 | 4  22  44  19  4  7 | 0  26  30  22  14  8 | 4  23  32  15  15  11 | 1  20  31  19  16  13 | **<0.0001** |  | | | | | | |
| Number of dental extractions that you do or refer each month  0  1-5  6-10  11-15  16-20  >20 | 0  25  30  13  17  15 | 1  38  30  9  10  12 | 0  33  33  19  15  0 | 0  38  8  10  4  10 | 4  66  23  4  2  0 | 1  33  30  11  13  12 | **<0.0001** |  | | | | | | |
| **Dentist individual characteristics** | | | | | | | | |  |  |  |  |  |  |
| Gender  Male  Female | 85  15 | 87  13 | 69  31 | 82  18 | 55  45 | 82  18 | **0.001** |  | | | | | | |
| Race  White  Black or African American  American Indian  Asian | 94  4  1  1 | 94  4  0  2 | 90  7  0  3 | 80  4  2  14 | 100  0  0  0 | 93  4  0  3 | **0.005** |  | | | | | | |
| Year of graduation from dental school  Before 1974  1974-1983  1984-1993  1994 or later | 19  39  24  18 | 19  43  22  16 | 0  37  32  31 | 4  26  31  39 | 13  38  22  27 | 16  38  24  22 | **0.004** |  | | | | | | |

- All statistical tests were performed using the chi-square test or two-sample t-test. p<0.05 is significant
